# Personalized workflows in reconstructive dentistry—current possibilities and future opportunities

**DOI:** 10.1007/s00784-022-04475-0

**Published:** 2022-03-30

**Authors:** Tim Joda, Nicola U. Zitzmann

**Affiliations:** Department of Reconstructive Dentistry, University Center for Dental Medicine Basel (UZB), University of Basel, CH-4058 Basel, Switzerland

**Keywords:** Personalized dental medicine, Digital transformation, Artificial intelligence, Augmented reality, Tele-dentistry, Prosthodontics

## Abstract

**Objectives:**

The increasing collection of health data coupled with continuous IT advances have enabled precision medicine with personalized workflows. Traditionally, dentistry has lagged behind general medicine in the integration of new technologies: So what is the status quo of precision dentistry? The primary focus of this review is to provide a current overview of personalized workflows in the discipline of reconstructive dentistry (prosthodontics) and to highlight the disruptive potential of novel technologies for dentistry; the possible impact on society is also critically discussed.

**Material and methods:**

Narrative literature review.

**Results:**

Narrative literature review.

**Conclusions:**

In the near future, artificial intelligence (AI) will increase diagnostic accuracy, simplify treatment planning, and thus contribute to the development of personalized reconstructive workflows by analyzing e-health data to promote decision-making on an individual patient basis. Dental education will also benefit from AI systems for personalized curricula considering the individual students’ skills. Augmented reality (AR) will facilitate communication with patients and improve clinical workflows through the use of visually guided protocols. Tele-dentistry will enable opportunities for remote contact among dental professionals and facilitate remote patient consultations and post-treatment follow-up using digital devices. Finally, a personalized digital dental passport encoded using blockchain technology could enable prosthetic rehabilitation using 3D-printed dental biomaterials.

**Clinical significance:**

Overall, AI can be seen as the door-opener and driving force for the evolution from evidence-based prosthodontics to personalized reconstructive dentistry encompassing a synoptic approach with prosthetic and implant workflows. Nevertheless, ethical concerns need to be solved and international guidelines for data management and computing power must be established prior to a widespread routine implementation.

## Introduction

People see themselves as individuals and want to be treated as such in everyday life. It is not for nothing that we look for individually tailored clothing that expresses a personalized style, play with online configurators in the automobile industry to create a unique car, or just simply desire likes on the various social media platforms for individual expression. Being different from the others—personality—is *en* *vogue* today [1]. On the other hand, a certain degree of conformity and standardization ensures safety through increased comparability and process simplification, along with the possibility of scalability to provide specific goods and services to a widespread population. This principle from the field of economics is also relevant to evidence-based (dental) medicine [2].

*When it comes to our own body, we want an individual consultation and the necessary medical therapy tailored to it. What does personalized (dental) medicine mean in this context?*

Personalized medicine is about providing a treatment that is as individualized as the disease with specific signs and symptoms. The approach relies on identifying genetic, epigenomic, and clinical information that allows the breakthroughs in the understanding of how a patient’s unique genomic portfolio makes them vulnerable to certain diseases. Each patient is to be treated with comprehensive consideration of individual circumstances using multi-disciplinary channels, beyond the solely functional aspect of disease diagnosis. This also includes the continuous adjustment of therapy to keep pace with the progress of medical knowledge plus advances in computer technology [3, 4].

Big Data is the twenty-first century game changer in medicine [5]. Digital health data is collected everywhere and at all times specifically, in the context of examinations and treatments by professionals in the healthcare industry as well as the insurance business, but also non-specifically through personal health apps, social media, and other devices that make up the Internet of Things (IoT). Due to the rapid progress in information technology, completely new approaches in dental medicine are feasible today [6].

A key technology in the future of healthcare is artificial intelligence (AI). AI has the potential to positively influence several areas of dentistry [7]. Currently, the main application of AI is on radiological diagnostics [8]. Using AI, dental imaging is quantified, analyzed, automatically compared to the defined normal, and then visualized [9]. Findings can be linked with patient-related health information in order to provide a personalized solution—so-called *Anatomical Intelligence*. Therefore, AI could enable much more precise diagnostics; and based on this, AI algorithms could identify and suggest the right therapy choice, by linking the specific diagnostic profile of an individual patient to the available treatment options [10]. AI could also be used to automatically monitor particular patient groups post-treatment. As well as applying AI to an individual patient to provide personalized oral healthcare, the technology could be used to analyze entire populations to derive precise solutions [1]. For example, a defined cluster of included patients within a certain area could be analyzed to see how many of these patients have specific dental diseases, to identify their risk profiles, and to understand what the costs to the system would be. AI could then stratify patient groups to interpret complex information and reveal insights into the specific needs of those patient groups or to predict potential future scenarios [2].

Traditionally, dentistry has lagged behind general medicine in the development and integration of new technologies. Therefore, the question arises, what is the status quo of precision dentistry, and here especially in the discipline of reconstructive dentistry (prosthodontics)? The focus of this review is to provide a current overview of personalized workflows in reconstructive dentistry and to highlight the disruptive potential of novel technologies in dental research and routine practice. In addition, the possible impact on society of these technologies, including ethical concerns, is critically evaluated.

## Discipline of reconstructive dentistry

The overall goal in dentistry is the maintenance of oral health and the prevention of tooth loss. The dental discipline of prosthodontics comes into play when there has been loss of teeth and adjacent tissue in order to achieve functional and esthetic rehabilitation including long-term stability, comprising fixed and removable prostheses retained on teeth and/or dental implants with or without mucosal support [11].

### Prosthetic complexity

Several patient-related factors must be taken into account in reconstructive dentistry; thus, the individual intraoral situations are not directly comparable. Diagnostics and therapy are holistic and include multiple variables, such as general health conditions, extend of the restoration, and not at least the number of existing abutment teeth, which is highly variable among patients. Considering 28 teeth, there are tens of billions of possible combinations of missing teeth and related distribution of possible abutments [12]. This vast number of potential options makes the planning and clinical execution of prosthodontic therapy a demanding and complex specialty that requires a knowledgeable and experienced reconstructive team based on the prosthodontist and the dental technician [13]. In other dental disciplines, such as restorative dentistry, endodontics, or periodontology, treatment needs can be very focused, and standard operating procedures (SOPs) applied with a clearly defined sequence of therapy steps following the principles of evidence-based dentistry [14]. In contrast, reconstructive dentistry is per se a highly personalized discipline and uniform SOPs are hard to implement—except for situations that are directly comparable, such as complete edentulous patients [15]. In the dentate patients, the prognostic assessment of each abutment tooth is influenced by prosthodontic, periodontal, and endodontic aspects, and the outcome of a complex prosthodontic rehabilitations can be affected by any of these factors. As long as a single-tooth restorations is planned in an intact arch, an abutment tooth with a questionable prognosis might be accepted, while multiple risk factors of a tooth intended as an abutment for a fixed dental prosthesis (FDP) expose the entire restoration to a higher risk [12].

### Economic driver

In addition, the selection of the reconstructive treatment option highly depends on the payer’s modality and related country-specific dental insurance system [16]. If a health policy system only pays for certain forms of treatment, the risk may be that patients and clinicians will be tempted to choose or perform only that particular therapy, even though another solution might be more appropriate from a dental point of view in that specific case. In these situations, clinical decision-making is no longer absolutely free and rather determined by external factors [17]. National differences in routine oral healthcare and dental research exist that can also restrict the opportunity for implementing personalized workflows.

## Personalized reconstructive workflows

The main driver for successful treatment of (complex) cases in reconstructive dentistry is the level of experience with dental prostheses combined with the knowledge and skills of the clinician and technician in a team approach. Furthermore, it has to be emphasized that prosthodontics is a digitally triggered technical discipline [18]. Access to an up-to-date digital infrastructure, such as intraoral and laboratory scanning systems, computer-aided design, and computer-aided manufacturing (CAD/CAM), as well as 3D-printing, must currently be considered a basic requirement for high-quality prosthetics in order to facilitate complete digital workflows [19].

Increasing level of digitization in reconstructive dentistry may lead to disruptive innovations in diagnostics, treatment planning, and prognosis assessment. Moreover, the therapeutic spectrum will be significantly expanded in the future. Digitalization, thus, provides the basis for further development to enable personalized workflows built around standardized diagnostics for individualized therapy with reduced dependence on the individual skills of the prosthetic team members [20].

In addition to all the technical advances in manufacturing protocols, the consideration of functional parameters in complex prosthetic rehabilitation is still missing today. Ideally, the acquisition of functional parameters, such as chewing, swallowing, and motor properties of the tongue, lips, and corresponding forces, should be included in the context of personalized workflows. In the future, reconstructive dentistry must also integrate these functional components.

### Artificial intelligence (AI)

AI-based processes are seen as a key technology in medicine and dentistry [21]. AI systems empower personalized dental workflows by analyzing all health data gathered from an individual patient. This includes genomic, proteomic, and metabolomic information; using these data, AI systems can determine optimized treatment strategies and risk management on an individual basis [7, 22].

In addition to the high potential of automated radiological diagnostics to identify caries, endodontic lesions, and cysts, future AI systems will likely be able to determine the individual risk of tooth loss considering preventive measures like cleaning ability, caries activity, and access to therapy. So these predictions on tooth prognosis will be made based on the individual dental situation, and also accounting for multiple social and medical parameters supported by Big Data analyses [23]. Such AI applications will support clinicians in decision-making for patient-specific prevention and therapy [24]. Moreover, closer digital networking with medical specialties will facilitate higher levels of data sharing, required to develop and train such AI systems. As the amount of anonymized patient data grows, these AI systems will steadily gain precision in terms of prediction accuracy [25, 26].

A concrete example is AI-based virtual implant treatment planning following prosthetic-driven backward protocols. Today, special software ensures the planning of the 3D implant position using cone-beam computed tomography (CBCT) and the subsequent transfer of the virtual situation into reality. The so-called computer-aided implant dentistry (CAID) offers an additional tool that can achieve a precise and predictable treatment result tailored to the individual patient situation [27]. However, the software is complex and requires a certain level of experience and knowledge [28]. In the future, AI technology will allow automated preparation of CBCTs, such as image segmentation and visualization of anatomical landmarks, the inferior alveolar nerve, or maxillary sinus [29]. The pre-prosthetic setup will be automatically integrated by the AI system which will then generate correctly aligned suggestions for the 3D position of the implant including length, diameter, and abutment connection. However, this technology can only serve as a supplementary support-tool and the treatment plan must always be checked and verified by the prosthodontist. Nevertheless, such AI systems could significantly help clinicians in the future and reduce the barriers to entry for CAID, potentially making the technique more widely available [30].

### Augmented reality (AR)

Augmented reality (AR) has the power to visualize complex issues in 3D, for example, to create a virtual dental patient based on the superimposition of different medical images [31, 32]. In particular, the combination of AR with CAID seems to be a promising match. AR technology can simulate the pre-op treatment planning which can then be displayed virtually to the patient [33]. The patient can thus see in advance the planned therapy and the expected outcome. When patient-specific risk factors are also incorporated into this process, it becomes a highly personalized workflow. Working together with the prosthetic team, AR can then be used to evaluate whether the proposed treatment plan is feasible and will deliver the result that the individual patient desires. Furthermore, AR facilitates an intensive exchange among dental professionals in an interdisciplinary team approach using smart dental glasses [34].

### Tele-dentistry

Advances in IT technology have enabled remote support from other colleagues [35]. As previously mentioned, AR can help to visualize and to explain complex topics following multi-channels, e.g., with smart glasses for visualization, data transfer, and communication. Highly specialized dentists such as prosthodontists in one location can support distant general practitioners dealing with complex patient cases through digital media networking [36]. This is also a form of personalized workflow in prosthodontics, not on the patient side, but from the perspective of the healthcare provider [37, 38]. Tele-dentistry can also be beneficial for the patient by enabling follow-up examinations to be conducted remotely or for answering simple dental enquiries without the patient having to visit the dental practice [22]. It is estimated that these forms of tele-dentistry will become increasingly important in the future and that new business models will arise that impact all dental disciplines [2, 39].

### Dental education

Dental undergraduate education will benefit from personalized teaching methods [40]. Besides the acquisition of theoretical knowledge, dentistry is strongly influenced by motor skill training [41]. A classic example is training in tooth preparation in the simulation clinic prior to the treatment of real patients. While today the same curriculum is usually applied to all dental students, in the future, AI protocols could be used to generate specific practical exercises personalized to the specific needs of each student [42]. Intraoral optical scanning (IOS) can also be used to personalized general training in crown preparation [43, 44]. By optically capturing student’s prepared tooth, and digitally matching it to an ideal preparation within an education software, the student can objectively see his or her personal result and progress, while dental instructors focus on education instead of evaluation, which remains somehow subjective [45]. Theoretically, 24/7 training would be possible, which would save on personnel resources as well. In addition, it is possible to record how often and with what success a student has prepared a tooth during these general training exercises [46]. Building on these initial results, individual training protocols can be developed for each student to customize the teaching program and achieve the necessary motor skills using the tools and channels that these digital natives are comfortable with [43].

### Digital dental passport

For the prosthetic rehabilitation of lost hard and soft tissue structures to closely match the original patient situation, it would be beneficial if the original situation is available digitally, e.g., as an STL file (standard tessellation language) obtained from IOS. This dataset could be easily generated for each patient once the permanent adult teeth have matured and stored as a kind of “oral health recovery passport” encoded using blockchain technology [47]. Using such an individual passport, tooth position, shape, and color—including the occlusal relation and vertical dimension—could then be rebuilt to restore the patient’s individual situation. Considering future developments in manufacturing technology, advances will provide new possibilities via rapid prototyping, e.g., 3D-printing of bio-physiological materials for patient-specific dentine-enamel tooth structures and soft tissue [48].

In addition, the digital dental passport could be combined with facial scanning in order to create a complete virtual dental patient [32]. This would provide additional information regarding the lip-frame. The individual smile design of the patient could also be actively taken into account in motion, allowing esthetic analyses to be more precise and therapy protocols to be implemented in a predictable (and, if necessary, reproducible) manner. Ideally, these digital dental passports would be repeated every decade to account for the continuous aging process of the patients, analogous to how a national passport is regularly updated with a current photograph. Regular updates would also ensure that the digital dental passport includes the latest technology in terms of data acquisition as well as data formats.

This passport should also record all materials used for prosthetic reconstructions, comprising technical and clinical information, such as ceramics, alloys, acrylics, and cementation, as well as maintenance recommendations for oral hygiene measures and intervals. Particularly for dental implants it is of utmost importance to document the applied system to facilitate identification in case of complications or re-treatment. In this context, a few national implant registers are already established in several countries. The linkage of these implant registers with the patient-based digital dental passport would allow valuable epidemiologic analyses for personalized reconstructive workflows in the future—ideally with international access option [10].

## Societal impact and ethical considerations

The disruptive potential of AI and other technologies as a basis for generating personalized workflows in reconstructive dentistry is very high. Nevertheless, many question marks still exist before such techniques can be considered for routine implementation [5].

### Data management and computing technology

While developments in information technology seem to promise endless possibilities, there are considerable issues surrounding data management in terms of data collection, storage, sharing, and security that must be solved [6, 49]. The quality of AI is only as good as the quality of the data that is fed into it. Therefore, high-quality data that is compatible, interoperable, and handled with appropriate privacy and security regulations—which is definitely non-trivial in healthcare—is essential [50]. Moreover, the IT infrastructure must be robust and stable. The computing power enabling complex AI algorithms needs to be further developed, e.g., combining e-health data from different fields and sources. Today, AI can be used for simple tasks, but its application operating complex facts is still challenging. For the successful development of an AI model and consecutive implementation into clinical routine, acceptance and trust in the technology are important. Currently, still very few AI applications are (routinely) used in dentistry. The question of a wrong diagnosis and the resulting consequences for therapy weigh heavily. Verifying the accuracy of the AI algorithm is therefore a mandatory safety step. In general, three criteria must be considered for the development and implementation of an AI tool in healthcare: trust, consistency, and explainability.

### Responsibility and malpractice

It remains to be determined what will be the impact and the consequence of errors in diagnostics or treatment planning due to AI-based mistakes and misinterpretation of data? From an ethical point of view, it is therefore indispensable that the final decision must always be made by certified clinicians, and it is they who are responsible and not an AI algorithm. Deciding how to address these ethical issues on a national level will be challenging, and there is also a need to establish international regulatory guidelines [51, 52].

Personalized dental medicine will create manifold possibilities to improve the provision of dental care, the extent of which cannot be envisaged at present. From an ethical point of view, it must also be considered, what should be done with unexpected findings? If, for example, medically relevant diagnoses are derived from any genetic material, which could be an extracted tooth which serves as a bio-copy for the prosthodontist, must the patient then be informed [53]?

### Demographic development

In general, the changing demographics and aging of the population have increased the proportion of patients aged over 70 years who have medically multi-morbid disease involvement [54]. This has a direct impact on dentistry as well, and especially on the discipline of reconstructive dentistry: (1) tooth loss occurs at higher ages resulting in more complex prosthetic treatment; (2) older individuals have worse acceptance of (removable) prostheses; and (3) there is a higher risk of supplementary implants in medically compromised patients [55, 56]. For these reasons, it is obvious that networking with general medicine, and in particular with geriatrics, will become increasingly important for the prosthodontist in the future to deliver personalized workflows in reconstructive dentistry.

## Conclusions

**Fig. 1:**
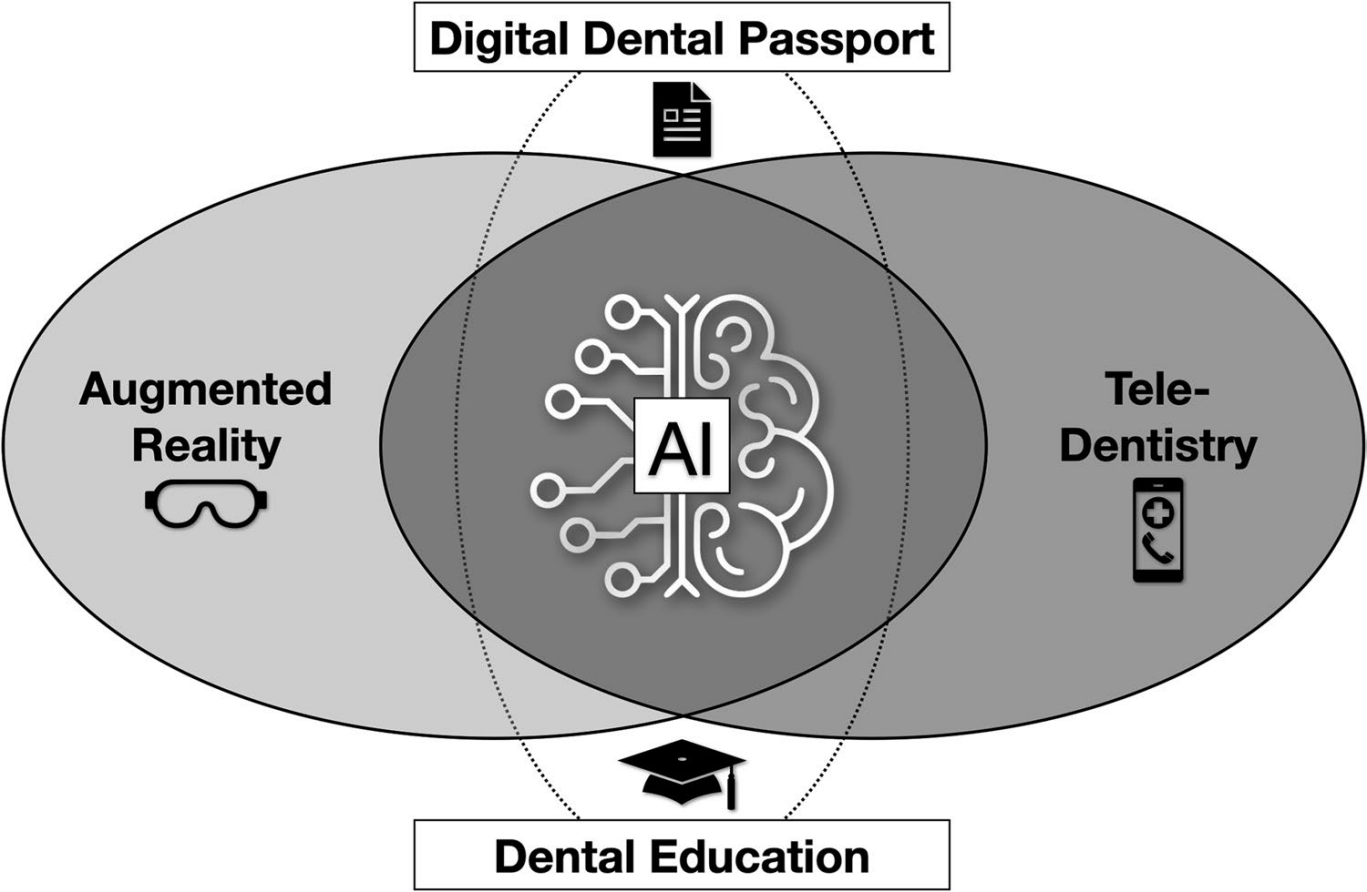
Artificial intelligence (AI) at the center of the interface of augmented reality (AR) and tele-dentistry as flanking technologies for use in dental education and treatment support as a digital dental passport including implant register

In the near future, AI systems will increase diagnostic accuracy as well as simplify treatment planning, and therefore contribute to the development of personalized reconstructive workflows by analyzing e-health data gathered from the molecular to the clinical level to improve decision-making on an individual patient basis. Dental education will also benefit from the introduction of AI systems for personalized curricula considering the individual skill levels of students, for example, in motor skill training. AR will facilitate communication between clinician and patients and improve clinical workflows through the use of visually guided protocols. Tele-dentistry will open up opportunities for remote contact among dental professionals, remote initial consultations with patients, and remote follow-up of patient condition using digital devices. Finally, a personalized digital dental passport including implant register could enable faithful prosthetic rehabilitation using 3D-printed dental biomaterials (Fig. 1) [7].

In this context, a new understanding of digital technologies must be acquired for successful application and permanent implementation in dental routine. In general, the traditional and established protocols from the analog era cannot be identically transferred to the digital processes. New standards are necessary for issues that did not exist in the analog world, such as regulatory aspects regarding the collection, storage, sharing, and analysis of acquired electronic patient data—and this is, of course, necessary for all dental disciplines, not only in reconstructive dentistry.

Overall, AI can be seen as the door-opener and driving force for the evolution from evidence-based prosthodontics to personalized reconstructive dentistry encompassing a synoptic approach with prosthetic and implant protocols for the maintenance of oral health and the rehabilitation of lost hard and soft tissue. The future focus should be to identify and develop the tools that can improve clinical decision-making. AI-based systems that can generate structured e-health data that is easily shared and transmitted, along with tools for interpreting and visualizing complex patterns, detecting abnormalities’ early warning signs, will all help the clinicians make the right decision at the right time. Several obstacles remain before truly personalized workflows can be implemented in routine practice. The allocation of oral healthcare research and funding opportunities should also focus on individualized healthcare delivery models that build the base of precision dentistry [57]. Therefore, public health policy and decision-making have to be transformed to reflect this new focus on personalized workflows and protocols to foster the general aim of diminishing healthcare disparities and improving oral health [58].

## Data Availability

N/A.
